# Transforming Agricultural Waste from Mediterranean Fruits into Renewable Materials and Products with a Circular and Digital Approach

**DOI:** 10.3390/ma18071464

**Published:** 2025-03-25

**Authors:** Antonella Castagna, Aouatif Aboudia, Amine Guendouz, Carmen Scieuzo, Patrizia Falabella, Julia Matthes, Markus Schmid, David Drissner, Florent Allais, Morad Chadni, Christian Cravotto, Julia Senge, Christian Krupitzer, Ilaria Canesi, Daniele Spinelli, Fadoua Drira, Hajer Ben Hlima, Slim Abdelkafi, Ioannis Konstantinou, Triantafyllos Albanis, Paraskevi Yfanti, Marilena E. Lekka, Andrea Lazzeri, Laura Aliotta, Vito Gigante, Maria-Beatrice Coltelli

**Affiliations:** 1 Department of Agriculture, Food and Environment, University of Pisa, 56126 Pisa, Italy; antonella.castagna@unipi.it; 2 Bioresources and Food Safety Laboratory, Faculty of Science and Technology of Marrakech, Cadi Ayyad University, P.O. Box 549, Marrakech 40000, Morocco; a.aboudia@uca.ma; 3 Agrobiotechnology and Bioengineering Center, CNRST-Labeled Research Unit (Agro Biotech-URL-CNRST-05 Center), Faculty of Science and Technology, Cadi Ayyad University, P.O. Box 549, Marrakech 40000, Morocco; a.guendouz@uca.ma; 4 Department of Basic and Applied Sciences, University of Basilicata, 85100 Potenza, Italy; carmen.scieuzo@unibas.it (C.S.); patrizia.falabella@unibas.it (P.F.); 5 Sustainable Packaging Institute SPI, Faculty of Life Sciences, Albstadt-Sigmaringen University, Anthon-Günther-Straße 51, 72488 Sigmaringen, Germany; matthes@hs-albsig.de (J.M.); schmid@hs-albsig.de (M.S.); drissner@hs-albsig.de (D.D.); 6 URD Agro-Biotechnologie Industrielles, CEBB, AgroParisTech, 51110 Pomacle, France; florent.allais@agroparistech.fr (F.A.); morad.chadni@agroparistech.fr (M.C.); christian.cravotto@agroparistech.fr (C.C.); 7 Department of Food Informatics and Computational Science Hub, University of Hohenheim, 70599 Stuttgart, Germany; julia.senge@uni-hohenheim.de (J.S.); christian.krupitzer@uni-hohenheim.de (C.K.); 8 Next Technology Tecnotessile Società Nazionale di Ricerca R.L., 59100 Prato, Italy; ilaria.canesi@tecnotex.it (I.C.); chemtech@tecnotex.it (D.S.); 9 Ecole Nationale d’Ingénieurs de Sfax, Université de Sfax, Sfax 3038, Tunisia; fadoua.drira@enis.tn (F.D.); hajer.benhlima@enis.tn (H.B.H.); slim.abdelkafi@enis.tn (S.A.); 10 Department of Chemistry, University of Ioannina, 45110 Ioannina, Greece; iokonst@uoi.gr (I.K.); talbanis@uoi.gr (T.A.); pyfanti@uoi.gr (P.Y.); mlekka@uoi.gr (M.E.L.); 11 Department of Civil and Industrial Engineering, University of Pisa, 56122 Pisa, Italy; andrea.lazzeri@unipi.it (A.L.); laura.aliotta@unipi.it (L.A.)

**Keywords:** circular economy, biobased materials, perishable fruits, plant waste residues, biocomposites, extraction, bioconversion, insects, digitalization

## Abstract

The Mediterranean area is one of the major global producers of agricultural food. However, along the entire supply chain—from farming to food distribution and consumption—food waste represents a significant fraction. Additionally, plant waste residues generated during the cultivation of specific fruits and vegetables must also be considered. This heterogeneous biomass is a valuable source of bioactive compounds and materials that can be transformed into high-performance functional products. By analyzing technical and scientific literature, this review identifies extraction, composite production, and bioconversion as the main strategies for valorizing agricultural by-products and waste. The advantages of these approaches as well as efficiency gains through digitalization are discussed, along with their potential applications in the Mediterranean region to support new research activities and bioeconomic initiatives. Moreover, the review highlights the challenges and disadvantages associated with waste valorization, providing a critical comparison of different studies to offer a comprehensive perspective on the topic. The objective of this review is to evaluate the potential of agricultural waste valorization, identifying effective strategies while also considering their limitations, to contribute to the development of sustainable and innovative solutions in Mediterranean bioeconomy.

## 1. Introduction

The Mediterranean region is among the most vulnerable areas in the world, significantly affected by climate change, biodiversity loss, water scarcity, and land degradation.

These challenges are exacerbated by inefficient resource use, particularly food waste, which is a significant concern. As a region that produces a wide variety of agricultural products, the Mediterranean diet—rich in fruits, vegetables, olive oil, cereals, legumes, nuts, and moderate amounts of fish and dairy—has become an important part of its culture and economy [1]. However, along the entire food chain from farm to fork, a considerable amount of waste and by-products are generated, representing both an environmental challenge and an untapped resource [2].

In developing countries, losses primarily occur in the early stages of the food chain, with around 40% of losses happening during post-harvest and processing stages. In contrast, in industrialized nations, food waste is more prevalent in later stages, particularly at retail and consumer levels [3]. In the European Union, it is estimated that 179 kg of food is wasted per capita annually, with households accounting for about 42% of total food waste. Similarly, the Near East and North Africa (NENA) region faces high levels of food loss, particularly at early stages of the food chain, due to environmental factors, inadequate storage, transport, and packaging infrastructure [4].

Given the high volumes of food waste generated in the Mediterranean, it is essential to improve the efficiency of the entire food chain. Food waste of perishable fruits is a major problem worldwide, with social, economic, and environmental repercussions. According to the Food and Agriculture Organization of the United Nations (FAO), approximately 1.3 billion tons of food are lost or wasted each year worldwide, representing one third of total global food production [5]. Furthermore, food waste, including that of perishable fruits, contributes to about 8 to 10% of global greenhouse gas emissions. This is also accompanied by the useless loss of natural resources, namely water (around 280 L of water is necessary to produce 1 kg of strawberries), arable land, and energy consumed for production and transport [6].

In several countries, food waste may account for nearly half of the total solid waste produced [7]. The major techniques commonly employed to treat food waste include anaerobic digestion, composting, incineration, sewage, and landfilling. It has been observed that these wastes are primarily disposed of in landfills, as this is the easiest and cheapest waste treatment method. However, this practice leads to significant land, air, and sometimes water pollution [8]. Approximately 7% of global greenhouse gas emissions are a consequence of food waste landfilling. Nearly 70.5% of the waste generated from the food processing sector and 50% of the waste collected from the wholesale and retail sectors are incinerated. In the food service sector, 21% of the waste is incinerated, while 54% is dumped in landfills. Additionally, 33.4% of the food waste from households is incinerated, and 27.5% is dumped in landfills. Thus, landfilling remains widely adopted [9]. Another important point is that plant waste residues are sometimes managed by burning on the fields [10]. Adopting alternative biocircular methodologies that valorize functional molecules, biopolymers, and biobased materials from these wastes is essential to address the concerns associated with current waste management practices. These approaches can help reduce pollution, lower greenhouse gas emissions, and promote a more sustainable and eco-friendly system.

The European Union and the United Nations have recognized the importance of tackling food waste, with the UN’s Sustainable Development Goal (SDG) 12.3 focusing on reducing food waste and losses. Research efforts are supported by EU initiatives and Mediterranean partnerships [11], such as the Partnership for Research and Innovation in the Mediterranean Area (PRIMA), which promotes sustainable solutions in water, energy, and food sectors.

**Figure 1:**
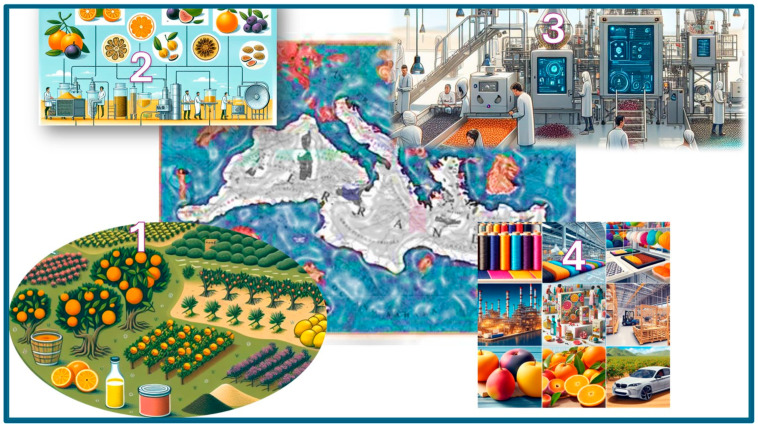
Main topics treated by the present review: (1) identify Mediterranean fruit by-products, (2) review processing technologies, (3) assess the role of digitalization, and (4) investigate high-value applications for sustainable development in the region.

This review aims to explore the issue of food waste in the Mediterranean region, specifically focusing on agricultural by-products such as fruit waste. The review will address the following objectives: (i) identifying the main sources of Mediterranean fruit by-products and waste; (ii) reviewing the current technologies available for processing these by-products; (iii) assessing the role of digitalization in advancing these technologies, and (iv) investigating high-value applications for these materials to support sustainable development in the region (Figure 1).

The potential novelty of the approaches presented in this review lies in their focus on valorizing agricultural waste using affordable, eco-friendly technologies, offering valuable by-products that contribute to reducing environmental impacts while promoting the circular economy [12].

Furthermore, this review will briefly highlight traditional methods of waste management, as they are still relevant in the short term, and examine the evolution of waste treatment and reuse in the context of modern technological advancements. The findings from this review aim to contribute to advancing research on sustainable waste management practices and promoting new opportunities for innovation in the Mediterranean food and agricultural sectors.

As a premise to guide the reader through the following paragraphs, it is important to underline that this review follows a structured methodology aimed at providing a comprehensive understanding of the Mediterranean region’s fruit by-products and waste. First, a thorough literature review was conducted by the multidisciplinary group of researchers co-authoring this work by collecting data focusing on recent studies related to Mediterranean food waste, its sources, and management strategies. Then, findings were harmonized in an interactive and collaborative way on the Microsoft OneDrive document sharing platform (https://www.microsoft.com/en-us/microsoft-365/onedrive/online-cloud-storage, accessed on 10 October 2024). The review specifically targets key waste streams from fruits like olives, citrus, and nuts, which are significant to the region’s economy. Some studies about non-Mediterranean plant waste are also sporadically mentioned in the review as examples of valorization of locally available raw materials in other geographical contexts. This review also examines the available technologies for processing these by-products, particularly innovations in recycling, upcycling, and the role of digital technologies in improving waste management efficiency. Finally, the review takes a multidisciplinary approach, integrating perspectives from fields such as agriculture, environmental science, and economics to provide a broader understanding of the challenges and opportunities. It stresses the importance of collaboration, strategic investments, and innovation for sustainable solutions and high-value applications for agricultural waste in the Mediterranean area.

## 2. Agricultural Waste of the Mediterranean Area

### 2.1. Perishable Fruits

Perishable fruits, such as fresh leafy greens and soft fruits, characterized by their rapid post-harvest deterioration, require specialized packaging to extend their shelf life. Typically, fruits with a rigid peel are packaged in flexible nets, while those with softer, thinner skins are placed in rigid trays often sealed with a film. Highly perishable fruits like berries lack protective skin, whereas fruits with tougher peels, like citrus, are less perishable but yet still contribute significantly to waste and by-products. Citrus fruits represent a major part of Mediterranean production. In the two-year period 2009–2010, 1.066 million hectares, that is, 12.2% of the world surface area, and a production of 22.5 million tons, that is, 18.3% of the world supply [13]. In 2023–2024, citrus production showed a significant increase and reached nearly 24.6 million tons [14]. Globally, it is estimated that around 30–40% of citrus fruits produced are lost or wasted, either due to post-harvest losses (damage during transport, inadequate storage) or by consumers (fruit not consumed in time). During 2023, Italy produced around 3 million tons of citrus fruits, which led to an estimated 700,000 to 800,000 tons of waste by-products [15].

The citrus processing industry alone globally generates an estimated 110 to 120 million tons of waste annually, posing significant challenges for waste management and environmental sustainability. Improper disposal of citrus waste can result in the pollution of land and water resources, contributing to soil deterioration and groundwater contamination. Moreover, the high organic content of this waste makes it prone to rapid decomposition, leading to the release of greenhouse gases if not managed properly. Enhancing the shelf life of citrus fruits could serve as an effective strategy to reduce fruit waste due to decay. Among citrus fruits, mandarins and tangerines require specific attention because, despite having a tough outer peel similar to other citrus species, this peel is reported to be the least resistant if compared with that of orange, lemon, and pummelo [16].

**Figure 2:**
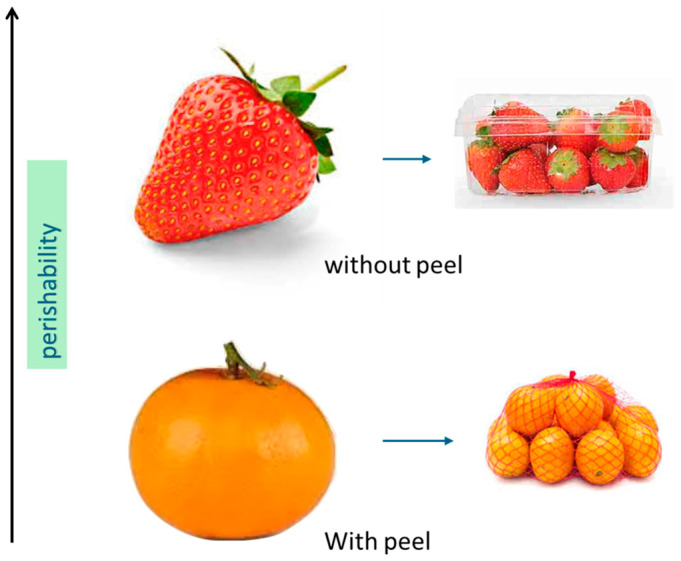
Examples of representative perishable fruits and their related packaging.

Fruits without peels need to be packed in rigid trays due to their softness. Strawberries, being highly perishable (Figure 2), have been the focus of numerous research activities aimed at developing and applying materials and methodologies to enhance their durability [17,18]. This fruit is widely diffused in the Mediterranean area and is appreciated for its pleasant taste and richness in antioxidants, vitamin C, and anthocyanins, making it an excellent source of these valuable beneficial compounds, but however, its elevated water content (approximately 90%), associated with the thin peel and the rapid decrease in flesh firmness during maturation, negatively affects its shelf life and makes this fruit easily susceptible to spoilage [19].

It is estimated that around 30–40% of strawberries are lost within the supply chain from field to consumer. At the household level, strawberries rank among the most wasted fruits, due to their rapid deterioration after purchase. Studies conducted in Europe and North America indicate that approximately 15–20% of purchased strawberries are discarded before consumption. Generally, fresh strawberries stored at temperatures between 0 and 4 °C last only 2–5 days. Indeed, their tender and juicy texture, coupled with a thin skin, makes them particularly vulnerable to mechanical damage and fungal infections, notably those caused by *Botrytis cinerea* [20], responsible for gray mold. Though strawberries are not climacteric fruits, they continue to respire after harvest, which accelerates their ripening and deterioration.

Another important fruit of the Mediterranean area is the *Phoenix dactylifera* date [21]. Dates are renowned for their high nutritional value and can be consumed either fresh or dried. Dried dates, thanks to their low water content and high sugar levels, have a longer shelf life compared to fresh fruits. However, if not stored properly, dates could dry too much and become less enjoyable. Moreover, depending on the storage conditions and their moisture content, dates are susceptible to fermentation, mold development, and mycotoxin contamination [22]. Furthermore, a considerable proportion of dates are rejected in the market due to their appearance or size, despite remaining perfectly edible.

Fruit-derived food waste remains a challenge to overcome. To tackle this issue, it is essential to implement initiatives aimed at improving supply chain management and raising consumer awareness. Moreover, there is a need to innovate new concepts of antimicrobial food packaging to reduce food waste, especially that of perishable fruits, to increase their shelf life.

### 2.2. By-Products of the Food Chain

#### 2.2.1. Fruits Shells and Stones

Nut shells constitute an abundant and underutilized waste from the agro-food sector. In terms of global production, almonds represent the highest share (31%), followed by walnuts (21%), cashews (17%), and hazelnuts (12%). Overall, the production value of nuts has increased significantly and steadily over the past decade, averaging USD 1.9 billion per year and reaching USD 35.6 billion for the 2019/2020 season [23]. As a result of nut industrialization processes, substantial amounts of by-products and residues are generated annually, most of them being either stored, burnt as fuel for heaters, or discarded as non-valued waste [24]. Shells represent the most abundant residue that should be valorized, considering the principles of circular economy. For example, argan shells, specific to the Moroccan territory, represent 52% of the co-products generated by the argan oil process. Almond, walnut, and argan shells are composed of cellulose, hemicellulose, lignin, and other bioactive compounds [25]. Many applications of nut shells have been explored. For instance, it has been reported that almond shells can be reused in the industry itself as biofuel, but they can also have structural functions. Indeed, they are used as organic inclusions in ceramic bodies [26]. Due to their lignocellulosic composition, applications to produce wood-based composites were explored. In addition, almond shells have been pointed out for environmental applications as heavy metal adsorbents in wastewater, soil amendments in the form of biochar, and for the preparation of activated carbon. Likewise, walnut shells and carbon-based materials development as adsorbents have been explored for eliminating heavy metals, pesticides, and other dangerous organic compounds, and synthetic industrial colors [27]. The research on the valorization of food and agricultural wastes through their transformation into adsorbent materials, such as biochar and activated carbon, demonstrates significant potential for addressing environmental challenges. Li et al. [28] explored the use of eggshells and oyster shells to enhance activated sludge-based biochar for phosphate removal, highlighting the effectiveness of calcium-loaded biochar as an efficient and low-cost adsorbent. Zheng et al. investigated the competitive adsorption of metal cations (Cu(II), Cd(II), and Ni(II)) using EDTA-modified biochar derived from peanut shells, revealing the complex interactions between these metals and the adsorbent materials. Zhuang et al. [29] applied supercritical carbon dioxide pretreatment to walnut shell waste to produce porous biochar with improved adsorption properties for dye removal, demonstrating a significant increase in adsorption capacity and surface area. Zhang et al. [30] studied apricot kernel shell biochar, emphasizing its potential for pesticide residue removal, specifically atrazine, and revealing its high adsorption capacity, particularly at higher preparation temperatures. Nursiah et al. [31] focused on cocoa shell-derived biochar for mercury ion adsorption, proving its efficacy as an environmentally friendly adsorbent material.

Regarding argan shells, in addition to their use in the production of purifying and adsorbent material, some studies have explored new ways of recovering the co-products (pulp, husks, and oil-cake) by the development of new natural, biodegradable, and non-toxic agro-composites and the production of four energy carriers (biodiesel, bioethanol, biohydrogen, and biomethane) by exploiting all argan co-products with 0% waste [32,33,34,35].

Fruit stones are significant by-products in the fruit processing industry, posing disposal challenges. The word “stone” typically refers to the hard seed inside fruits like dates, cherries, or peaches. It can also be referred to as a “pit” or “endocarp” [36]. For instance, date stones account for 7% to 30% of the total fruit weight, with their size varying across cultivars. Typically, date stones weigh between 0.6 and 1.69 g, with diameters ranging from 0.58 to 1 cm and lengths from 2.9 to 3.15 cm. Traditionally, date stones have been used as animal feed and soil fertilizers, as well as natural low-cost fillers [37]. Recently, they have gained attention for their use in the production of functional foods, including muffins, cookies, gluten-free products, cakes, coffee, bread, biscuits, cooking oil, bio-oil, cosmetics and pharmacology, and dietary supplements [38]. Although fruit stones are typically produced and discarded at the household level, the shift in consumer preferences towards healthier, ready-to-eat options has increased the sale of pre-cut fresh fruits. This trend will generate significant amounts of selectively collected waste stones, which could be utilized for new bioeconomy initiatives.

#### 2.2.2. Pomaces

Pomace, or “marc” is a by-product obtained after juice or oil extraction, essentially from apples, citrus, grapes, and olives. According to Gouw et al. [39], only 20% of the generated apple pomace is valorized. When citrus fruits are processed into industrial citrus juice, nearly half of their volume is wasted, and the enormous amount of citrus pomace causes serious ecological problems [40]. Improper management of citrus residues, such as pomace, generated by the agro-fruit industry can lead to significant environmental consequences and economic losses. Citrus pomace contains a significant number of organic compounds that, if not properly treated, can cause environmental issues like soil and water pollution and promote the proliferation of undesirable microorganisms. However, citrus residues also contain valuable compounds, including essential oils, polyphenols, fibers, and pectins, which can be extracted and utilized in the food, cosmetic, and pharmaceutical industries [41]. Thus, the absence of its valorization represents a loss of economic opportunities. For instance, fruit pomaces have been used to create biocomposites and films (blueberry/cranberry/grape mixture, acai berry, apple, blueberry, date, rambutan, durian, olive, among others) for packaging applications [42]. Otherwise, fruit pomaces can serve as biofuel, biofertilizer, biochar, and in biopolymer production. Furthermore, they had applications for food, namely in bakery, meat, dairy, and extruded products, but also in the confectionery industry (sweets and candies). They had also been used for the extraction of pectin, for obtaining valuable products by biotransformation, and as a source of fiber and fillers [43].

The composition of pomace varies by fruit and may include skins, flesh, seeds, stems, and residual pulp. For instance, olive pomace, whose typical composition [44] is shown in Table 1, which is a mixture of olive pulp and stones, is a source of functional compounds such as polyphenols, tocopherols, sterols, and others. This by-product has applications in foods as an ingredient (e.g., edible oils, pasta, fish burgers), for improving food packaging, or for shelf-life prolongation. Olive pomace also holds significant potential as a functional animal feed additive [45].

Finally, applications in the cosmetic industry and soil amendments are also significant. In the context of widely abundant waste from fruits, another important waste consists of the grape pomace, obtained during wine production, widely diffused in Southern Europe. Approximately 50% of grape pomace is made up of grape peels (GP), depending on the grape variety and pedoclimatic conditions [46]. There are some studies revealing the possibility to create value-added products by incorporating grape by-products in bakery products or pasta [47].

Nevertheless, the most abundant by-product of the winemaking process is the grape pomace, resulting after the grape pressing. Since 20–25% of the weight of processed grape remains as pomace. Globally, about 10.5–13.1 million tons per year of grape pomace are produced [48].

#### 2.2.3. Peels

Fruit peels, often considered agricultural waste, possess significant potential for valorization. Citrus peel waste generated by the citrus-processing industry accounts for up to 50% of the total fruit weight, which is a significant fraction compared to peel waste from other fruits [49]. Citrus fruits, such as oranges, grapefruits, lemons, limes, and tangerines, hold significant global importance due to their widespread cultivation and consumption. As a staple in many diets and a key ingredient in various products, the demand for citrus fruits continues to rise. However, this popularity comes with a substantial environmental cost. A significant portion of the fruit, typically 40–60% of its mass, is discarded as waste during processing. This waste primarily consists of peels, seeds, and pulp, which are often underutilized and disposed of in ways that can lead to environmental harm. The citrus processing industry alone generates an estimated 110–120 million tons of waste annually, presenting severe challenges in terms of waste management and environmental sustainability. Improper disposal of citrus waste can lead to soil and water pollution, as well as groundwater contamination. Poor management of this waste can also release greenhouse gases due to rapid decomposition. Citrus waste mainly consists of membranes, peels, and pulp and is rich in sugars, fibers, organic acids, proteins, essential oils, and polyphenolic compounds. Its composition varies depending on the citrus species, variety, and harvesting season. Waste makes up 5–70% of the fruit, with peels, internal tissues, and seeds being the main components [50].

Phenolic compounds, flavonoids, phenolic acids, and coumarins, having antioxidant and antimicrobial activity, can be extracted from fruit waste peels [51]. Citrus pomace derived from the industrial processing of juice mostly consists of pectin, cellulose, hemicellulose, and simple sugars. Pectin is a natural constituent of all terrestrial plants that are particularly abundant in the primary cell walls of fruit and vegetables. The chemical composition and structure of pectin is very complex and depends on the source, extraction methods, plants, storage, and maturity of the raw plant source. Nevertheless, it is generally accepted that the pectin structure consists of heterogeneous polysaccharides with three main structural domains covalently linked one to another, the homogalacturonan, the xylogalacturonan, and the rhamnogalacturonans regions [52]. At a neutral pH, pectin, bearing carboxylic groups on its repeating polymeric units, is negatively charged, which enhances its water solubility. However, as the pH decreases, the negative charge on pectin decreases, and it can become water-insoluble, contributing to its gelling ability. Commercially, both citrus and apple pectin, which typically have a high degree of esterification and molecular weight, are commonly used as preservatives, gelling agents, and thickening agents in the food processing industry due to these properties. Cellulose can also be extracted from peels after a multistep process based on cleaning, grinding, alkaline treatment (to dissolve hemicellulose and partially remove lignin), bleaching, acid hydrolysis, purification, and drying [53].

#### 2.2.4. Plant Waste Residues

The cultivation of fruit plants generates substantial amounts of solid waste, mainly composed of primary residues (stems, leaves, and straw) resulting from the harvesting of annual plants or from the pruning of perennial species. Indeed, approximately 140 gigatons of biomass waste are produced annually from various crops, including fruits like bananas, oranges, grapes, and apples [54,55]. It is estimated that globally 11.2–16 million tons of vine shoots are produced each year.

Generally, this waste is managed through composting to enhance soil fertility, though burning remains a common practice in several countries. The most prevalent Mediterranean crops generate significant amounts of solid waste that could be better valued to obtain valuable products across various market sectors. For example, vineyards yield significant quantities of agricultural residues consisting of vine shoots and leaves. It can be estimated that globally 11.2–16 million tons of vine shoots are produced annually. Furthermore, the production of olive oil contributes to this issue with over 8 million ha of olive trees cultivated worldwide, especially in the Mediterranean basin. An average of three tons of pruning biomass per hectare is generated annually, making these residues a huge, low-cost, and unexploited source of energy [56] or chemicals.

Indirect residues from olive oil production include leaves accompanying the olive fruits to the mill, rich in phenolic compounds, which endow them with a high added value. Agricultural waste is mainly lignocellulosic biomass made up of three components (25 to 44.2% cellulose; 10.5 to 40.4% hemicellulose, and 21.7 to 44% lignin), along with bioactive compounds such as phenolic acids, flavonoids, anthocyanins, carotenoids, and vitamins with multiple antimicrobial and antioxidant properties [57,58]. These residues also contain minerals (5 to 10% of dry weight, mainly calcium, potassium, magnesium, and phosphorus), small amounts of proteins (2–5% of the dry weight), carbohydrates, and a high-water content (50–80%, varying with development stage and storage conditions). For example, strawberry plant debris contains flavonoids (quercetin, kaempferol), ellagic acid, and tannins, which play a defensive role against pests and pathogens [59]. Similarly, citrus plant debris are rich in flavonoids (naringin, hesperidin) and phenolic acids (caffeic acid, ferulic acid) and contain carotenoids as well [59]. Polyphenols extracted from plant debris are rich in flavonoids (quercetin), phenolic acids (ferulic acid, caffeic acid), and tannins. Green leaves and stems contain chlorophyll *a* and *b* that could be present in the waste material depending on the processing and storage conditions. Moreover, the stems and leaves of citrus species contain essential oils, rich in monoterpenes contributing to their characteristic odor, such as limonene, with antioxidant and antifungal properties, and linalool and geraniol, present in smaller quantities, with antimicrobial effects.

Unfortunately, pesticide residues may remain on plant debris after harvest, potentially being an obstacle for their harnessing, particularly for use in animal or human food. Nonetheless, agricultural waste has diverse applications, including the extraction of cellulose for uses in industry, agriculture, food packaging, etc. [60]. In addition, biotechnological techniques such as anaerobic digestion, fermentation, and composting can convert this abundant and free waste biomass into valuable biorefinery products [61].

Agricultural waste also serves as a source of food additives, due to the content of functional compounds such as peptides, carotenoids, and phenolic compounds. For instance, phenolic compounds from eggplant have been highlighted as a multifunctional food additive with antimicrobial, antioxidant, and coloring properties [62]. Olive leaves are commonly used in food products as natural additives to enrich them with bioactive compounds and extend shelf life through improved oxidation stability [63]. In addition, incorporation of these bioactive compounds in the formulation of coatings, films, and bioplastics can enhance the preservation and shelf life of foods [64,65].

## 3. Recovery by Extraction of Functional Molecules

### 3.1. Bioactive Compound Extraction and Purification

Among the fruits present in the Mediterranean area, the industrial processing of tangerines, dates, and strawberries generates substantial amounts of by-products, which are valuable sources of bioactive compounds [66]. Tangerine waste primarily comprises peel, pomace, and seeds, with the peel representing 50–55% of the fruit’s total mass [67]. Date processing results in various residues such as seeds, press cake, and cull dates (out-grade dates) [68]. Similarly, strawberry post-harvest processing yields significant by-products, including sepal, calyx, stem, and non-marketable portions of the fruit, which account for up to 20% of total production. These fruit wastes are rich in various compounds, including polyphenols, polysaccharides, carotenoids, and essential oils, which offer potential for diverse applications in nutraceuticals, pharmaceuticals, biomaterials, biorefineries, and cosmetic industries [69]. In recent years, the incorporation of natural bioactive compounds into coatings for paper and plastic substrates [70] or active packaging [71] has attracted considerable attention for their ability to improve food safety and quality by creating films with antioxidant, antimicrobial, and innovative color properties [72]. The extraction of bioactive compounds from agro-food waste is a major challenge due to their complex and heterogeneous chemical structures and their rapid perishability [73]. Food by-products are usually collected, separated, and dried before further use. However, in-situ processing and extraction could eliminate the need for drying. Moreover, high temperatures and prolonged exposure during conventional drying often lead to the degradation of relevant compounds, while alternative methods such as vacuum drying, freeze drying, fluidized bed drying, and instant controlled pressure drop drying (DIC) better preserve phytochemical contents [74]. Subsequently, by-products can undergo various pretreatments, such as physical (e.g., grinding), chemical (e.g., acid or alkali treatment), or biological conversion (e.g., enzymatic hydrolysis), to increase the extraction efficiency of target molecules; however, these methods may affect the purity and bioactivity of the extract (especially chemical treatments), so careful optimization is required [75].

**Figure 3:**
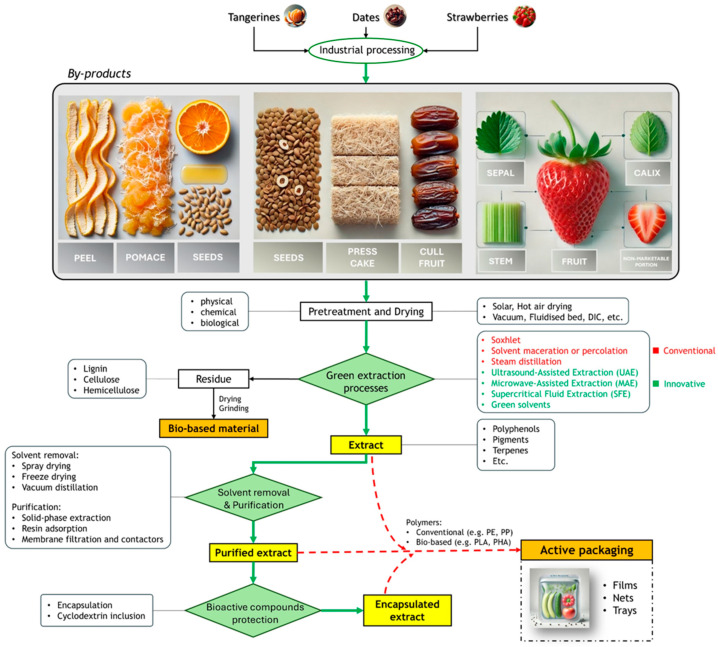
Valorization of Mediterranean fruit by-products for bioactive compound extraction and applications in active packaging.

Traditional extraction methods, such as Soxhlet extraction, solvent maceration or percolation, and steam distillation, are typically energy-intensive, time-consuming, and often rely on the use of hazardous solvents [76]. To overcome these challenges, green extraction processes have proven to be promising alternatives that offer higher yields, better product quality, and shorter extraction times, while minimizing the use of harmful solvents and reducing environmental impact. Innovative extraction technologies include ultrasound-assisted extraction (UAE), microwave-assisted extraction (MAE), supercritical fluid extraction (SFE), pressurized liquid extraction (PLE), and the use of green solvents [77]. After solid/liquid extraction, the solvent is typically removed to recover a solid extract: aqueous extracts are dried via spray drying or freeze drying, while volatile solvents are usually distilled under vacuum. In the case of Supercritical Fluid Extraction with CO_2_ SFE-CO_2_ and liquefied gas extraction (e.g., propane, n-butane), solvent-free products are directly obtained after extraction. However, a co-solvent like ethanol is often used in SFE-CO_2_ to enhance the solubility of more polar compounds [78]. In addition, extract purification, whether before or after solvent removal, is crucial for the isolation and concentration of the bioactive target substances. Common purification techniques include solid-phase extraction, resin adsorption, and membrane filtration [79]. Furthermore, liquid–liquid extraction through membrane contactors is gaining increasing attention due to its potential for large-scale applications [80]. Figure 3 shows a general process to produce active packaging using bioactive compounds derived from tangerine, date, and strawberry by-products.

Currently, the primary challenges to the widespread incorporation of natural bioactive substances in food packaging include their relative instability (e.g., thermal or oxidative) during polymer production or storage, the potential for undesirable sensory effects (e.g., strong aromas or flavors) [81], and their impact on packaging material properties, such as structural, mechanical, thermal, esthetic, and barrier characteristics [76]. Encapsulation offers a promising solution by protecting bioactive substances from degradation, increasing their stability, and prolonging their release. Polymers such as chitosan, gum, maltodextrin, pectin, and alginate are commonly used to retain these compounds and regulate their release under certain conditions, such as pH changes [82]. Cyclodextrins are another interesting alternative as they form inclusion complexes that protect bioactive compounds while controlling their release. Their use in industrial packaging is expected to increase due to their efficient and cost-effective encapsulation capabilities [81].

### 3.2. Applications of Extracted Molecules in High-Performance and Added-Value Functional Products

**Figure 4:**
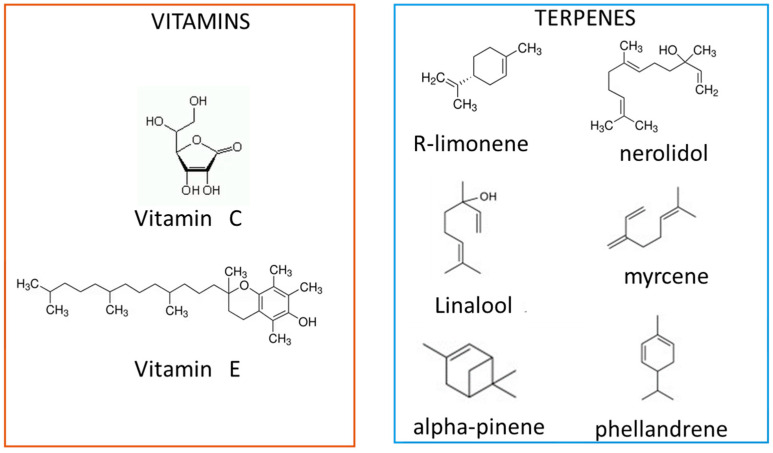
Chemical structures of some vitamins and terpenes present in fruits, trees, and their by-products.

In the various parts of plants, many natural compounds showing antioxidant activity were identified. These compounds can be categorized into vitamins (Vitamin C and E), terpenes, and polyphenols. Fruits and vegetables are rich sources of vitamin C [83]. In human nutrition, vegetable oils, nuts, and seeds are the main dietary sources of vitamin E, whereas fruits and vegetables are the primary source of vitamin C (Figure 4).

Terpenes (Figure 4) are present in essential oils, extractable from plants, and show both antioxidant and antimicrobial properties. They are organic compounds made up of isoprene, a five-carbon building block. Monoterpenes (C10) are cyclic or aliphatic molecules consisting of two isoprene units, sesquiterpenes (C15) and diterpenes (C20) of 3 and 4 isoprene units, while triterpenes (C30) and polyprenes have a higher molecular weight. Terpenoids, a class of secondary metabolites derived from terpenes, feature multiple cyclic groups and often contain oxygen. Volatile terpenes are thought to play essential roles in plant defense. Additionally, terpenes and terpenoids have applications in flavors, drugs, and fragrances. Terpenes are typically volatile or semi-volatile, whereas terpenoids can be non-volatile or semi-volatile due to the presence of other polar groups. High emission of terpenoids was detected by Iqbal et al. [84] in the peel of tangerine, followed by orange juice. These authors evidenced that R-limonene was the single predominant component in all the tested fruit samples with more than 90% abundance in all cases. α-pinene, β-phellandrene, and β-myrcene are major components of tree oleoresins, so they could be present in pruning waste of plants, whereas both linalool, which imparts a floral/citrus-like flavor, and nerolidol, providing a rose, apple, green flavor, were detected in cultivated varieties of strawberry [85].

**Figure 5:**
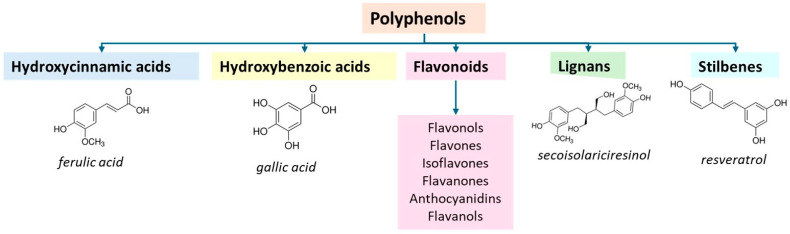
Classification of polyphenol compounds extractable from various parts of plants and fruits.

Among the most efficient antioxidants, we can mention the huge class of polyphenols (Figure 5), consisting of hydroxycinnamic acids, hydroxybenzoic acids, lignans, stilbenes, and flavonoids. The latter class consists of flavonols, flavones, isoflavones, flavanones, anthocyanidins, and flavanols. These compounds typically show antioxidant, antimicrobial, and anti-inflammatory properties [86]. Hence, they can be considered to design functional products to be used in packaging, pharmaceuticals, personal care, hygiene, and biomedical sectors. Interestingly, some colored compounds belonging to hydroxybenzoic, anthocyanins, and anthocyanidines, or flavanols, show the ability to modify their color depending on pH, so they were utilized in applications where a sensor of pH is required [87], as, for instance, in the packaging sector [88].

Polyphenols and terpenes are effective antimicrobial agents, either alone or in plant extracts and essential oils. Their antimicrobial action is mainly due to membrane disruption, altering membrane potential, and increasing permeability, which affects bacterial homeostasis. Compounds with high antimicrobial activity often share features like hydroxyl groups and conjugated structures. Additionally, water-extracted carbohydrate polymers from plants and lignin, found in lignocellulosic plant parts, exhibit antioxidant properties. Lignin, with applications in polymers, films, hydrogels, and stabilizers, also has antibacterial, anti-freezing, UV-barrier, and conductive properties [89].

#### 3.2.1. Antioxidant and Anti-Microbial Products

In active packaging and in the hygienic, protective, and personal care sectors, the use of natural active agents, such as essential oils or polyphenol-rich extracts, is currently of interest [90]. Among various natural substances, ferulic acid and rosmarinic acid have gathered attention because they are both antioxidant and antimicrobial [91]. It was reported that the incorporation of ferulic acid into ethylene vinyl alcohol through extrusion blending resulted in films with remarkable antioxidant and antimicrobial features against both Gram-negative and Gram-positive bacteria [92]. On the other hand, Ordoñez et al. [93] demonstrated that incorporating ferulic acid into polylactic acid (PLA) through casting and compression molding did not guarantee significant antimicrobial activity. However, Sharma et al. [94] reported that adding ferulic acid to a PLA/PBAT (polybutylene co-adipate terephthalate) blend conferred UV barrier properties and effective antimicrobial activity, thus limiting the growth of pathogenic bacteria on packaged food products. Kahya et al. [95] showed that chitosan films mixed with sage and rosemary extracts containing rosmarinic acid possess antioxidant properties and increased antimicrobial capacity compared to untreated chitosan. The data also revealed that rosmarinic acid prevents oxidative deterioration of the polymer film and migrates by diffusing into the food.

Another organic acid with interesting properties is 18β-glycyrrhetinic acid, obtained by hydrolysis of glycyrrhizic acid extracted from licorice. This acid is primarily used in pharmacological and cosmetic applications for its proven antimicrobial and anti-inflammatory activity [96]. Recently, nanostructured systems based on chitin nanofibrils and nano lignin encapsulating 18β-glycyrrhetinic acid have been prepared [97], which have shown cytocompatibility and anti-inflammatory activity when incorporated into PLA or deposited on the surface of PLA films [98]. Cicogna et al. [99] developed innovative films by integrating magnesium-aluminum layered double hydroxide (LDH)-based nanocarriers modified with functional molecules (rosmarinic acid [100], ferulic acid, and glycyrrhetinic acid) into a fully biobased poly(lactic acid)/poly(butylene succinate-co-adipate) (PLA/PBSA) matrix. The selected functional compounds are well known for their antioxidant, antimicrobial, and anti-inflammatory properties. Bioactive molecules can be gradually released from the nanocarriers over time, allowing for sustained and controlled delivery in various applications, such as active packaging or cosmetics.

Polyphenols were recovered from fruits or plant waste and can be used for developing innovative packaging solutions, both biobased and biodegradable. Polyphenols extracted from watermelon can be applied onto cellulose substrates like paperboards [101] or flexible veiled paper sheets [102], resulting in both cases in enhanced antioxidant and antimicrobial properties of the products for packaging and personal care applications, respectively. Lignin is also reported as a promising antioxidant and antimicrobial functional material to be used in packaging, considering also its UV light-blocking properties [103]. The incorporation of lignin into polymeric films has been achieved by solution casting, blown film extrusion, and compression molding. To attract industry to produce lignin-derived goods, cost-effective lignin valorization technologies should be better developed.

#### 3.2.2. Intelligent Devices

**Figure 6:**
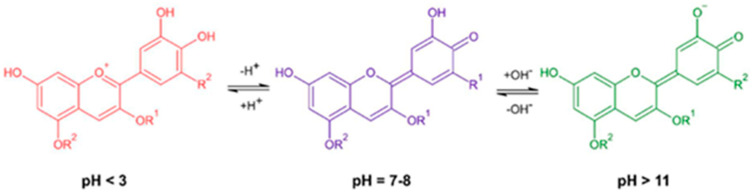
Structure and color of anthocyanins as a function of pH variations. R^1–2^ = _H or CH_3_.

Some molecules extracted from agricultural fruit by-products can be used as sensors in packaging and biomedical devices. The use of plant waste as a source of sensor molecules such as peels, roots, and leaves could be very promising. The concept of using waste instead of an edible source has gained popularity in recent years, mainly due to the ethical question of how food sources should be used. Indeed, grapes [104], mulberries [105], and blueberries [106] from wine and juice production, respectively, have been successfully used as a source for the manufacture of completely natural colorimetric indicators. The functioning of these devices is based on anthocyanidins. Their basic structure is composed of the C6—C3—C6 backbone called the flavyl cation, and, depending on the number and position of the hydroxyl and/or methyl ether groups, there are different types [107]. Although no more than 30 anthocyanidins have been identified to date, 90% of the anthocyanins present in nature are based on only six structures (30% are based on cyanidin, 22% on delphinidin, 18% on pelargonidin, and the remaining 20% on peonidin, malvidin, and petunidin). Depending on the hydroxylation and methylation of the flavonoid skeleton, the components range from orange-red (pelargonidin) to violet-blue (delphinidin). The coloration of anthocyanins changes reversibly when exposed to an environment with a different pH. Specifically, during protonation/deprotonation, the electronic structure is rearranged, and the total number of resonant electrons changes, and this causes color changes. At highly acidic pH (pH 1–3), the predominant tautomeric form is the flavilium cation, which has a red color (Figure 6). At pH values between 4 and 6, a mixture of balanced anthocyanins is formed, such as red-colored flavyl cations, purple or blue-colored quinonoidal bases, and yellow-colored chalcones, leading to an overall purple color. At pH values between 6 and 8, there is a further deprotonation of the quinonoidal bases with the consequent formation of more bluish quinonoid anions stabilized by resonance (quinoidal anion base). Finally, at pH 9, the color progressively changes to green and yellow tones due to further deprotonation of the molecule and the presence of the quinoidal dianion (green/yellowish green), chalcone (yellow), and carbinol [108].

The main challenge for the use of anthocyanins as pH indicators is their relatively low stability. Monomeric anthocyanins have been found to be extremely unstable and can be easily degraded to colorless or brown compounds, losing their ability to indicate pH. Structure also plays an important role in the stability of anthocyanins, with pelargonidin, cyanidin, and delphinidin being less stable than peonidin, petunidin, and malvidin. Furthermore, the stability of anthocyanins is influenced by environmental factors, including temperature, light, and the presence of other phenolic compounds, enzymes, metal ions, sugars, ascorbic acid, and oxygen. Specifically, their stability decreases with increasing pH, temperature, and sugar content, and their use is recommended at low temperatures, such as those of refrigerated products [109].

Choi et al. [110] developed a new colorimetric pH indicator film using agar, potato starch, and natural dyes extracted from the purple sweet potato, Ipomoea batatas. Both agar and potato starch are solid matrices used to immobilize natural dyes, anthocyanins. The ultraviolet-visible (UV-vis) spectrum of anthocyanin extract solutions and potato agar/starch films with anthocyanins showed color changes at different pH values (pH 2.0–10.0). Fourier transform infrared (FT-IR) and UV-vis spectra showed compatibility between agar extracts, starch, and anthocyanins. The color changes in the pH indicator films were measured using a colorimeter after immersion in buffers of different pH. An application test was conducted for potential use as a meat spoilage sensor. The pH indicator films showed changes in the pH and spoilage point of the pork samples, changing from red to green. Therefore, the developed pH indicator films could be used as a diagnostic tool for detecting food spoilage. Anthocyanidins extracted from fruits were also considered in the literature. For instance, Micó-Vicent et al. [111] used anthocyanidins extracted from pomegranate fruits to obtain materials suitable for intelligent packaging.

## 4. Recovery by Production of Composites

### 4.1. Production of Biocomposites and Their Properties

A biocomposite is a composite material composed of a matrix (resin) and reinforced with natural fibers. Biocomposites are widely applied across various industries, including furniture [112], the building [113], and automotive sectors [114]. These materials serve both structural and aesthetic purposes due to their high stiffness and low density. Additionally, biocomposites are valued in packaging [115] and personal care/cosmetics [116] for their enhanced biodegradability, affordability, and contribution to a circular economy [117].

Nutshell is a very abundant waste, and its valorization in the composites sector was considered by several researchers. For instance, Baiamonte et al. [118] investigated polylactide (PLA) biocomposites with hazelnut shells (HS) as fillers, assessing mechanical and rheological properties as well as biodegradability after composting and photo-oxidation. They found that fillers increased stiffness but reduced tensile strength and improved PLA’s UV resistance. Additionally, both fillers reduced compost degradation, as photo-oxidation accelerated the degradation process. Aliotta et al. [119] investigated the production by twin screw extrusion of composites consisting of a PLA matrix and a hazelnut shell powder (HSP) reinforcement. PLA-based composites containing hazelnut shell powder (HSP) were successfully produced at a semi-industrial scale, demonstrating enhanced mechanical properties compared to lab-scale composites. The investigation of these biocomposites indicated that it is possible to optimize the balance between incorporating a high content of HSP and achieving improved mechanical performance. In a recent study, the potential of HSP in 3D printing, showing that adding 10% HSP increased the density of 3D-printed specimens, has been explored [120].

The analysis of published studies on biocomposites containing nutshell powders, including walnut, almond, cashew nut, etc. [121,122,123], indicates that roughly half of the papers reviewed focus on biocomposites with a biobased thermoplastic matrix. Polylactic acid (PLA) is the most studied matrix, followed by thermoplastic starch and polybutylene succinate (PBS). This focus reflects a preference for renewable, compostable matrices that contribute to products with sustainable life cycles, which is particularly valuable in packaging and personal care applications. Conversely, the remaining half of the papers examine fossil-based thermoplastic and thermoset matrices, geared toward applications requiring greater durability, such as furniture and automotive components. Notably, WSP (walnut shell powder) has been more frequently studied in fossil matrices, ASP (almond shell powder) in biobased thermoplastic matrices, and HSP (hazelnut shell powder) exclusively in biobased thermoplastic matrices. However, literature suggests that micrometric powder from various nut shells can be suited to both long-lasting and short life-cycle applications.

Regarding other fruit by-products, a recent study utilized citrus pomace waste from a South Italian agricultural company as a source of pectin [124]. Pectin extracted from citrus pomace biomass using low concentrations of a mild organic acid, such as acetic acid, can be used to prepare biodegradable mulching films and biocomposites based on the water solutions of polysaccharides and cellulose fibers. These solutions are directly sprayable onto the soil and can form a protective geomembrane against the growth of spontaneous weeds that affect crop production. At the end of the cultivation period, the geomembranes can be buried into the soil where they are biodegraded by bacterial flora. Specifically, pectin was separated from the solid fiber fraction in the pulp, and these components were recombined into functional biocomposites. Additionally, bioactive molecules were isolated from the citrus pulp, with total phenolic content (TPC) and total flavonoid content (TFC) assessed. Aiming to create innovative biocomposite-based materials, the pectin was reinforced with varying amounts of lignocellulose fractions, also sourced from citrus waste after polysaccharide extraction, following a “zero waste” circular economy model. These biocomposites were characterized for their morphology and mechanical properties and were intended for use as biodegradable mulching systems for crop protection. Notably, the addition of citrus waste enhanced the biodegradation of bioplastic films, highlighting the potential of such agricultural by-products in creating sustainable and biodegradable materials.

In a study conducted in 2020, orange tree prunings were collected from cooperatives in Palma del Río, Córdoba, Spain, consisting of leaves and branches [125]. The biopolyethylene (BIO-PE) matrix used was suitable for injection molding. Maleic anhydride-modified polyethylene (MAPE) was used as a coupling agent to improve the bonding between the fibers and the matrix. For comparison, high-density polyethylene (HDPE) and glass fibers were also prepared using MAPE. Composites with 20% orange tree pruning fibers and different amounts of MAPE (0% to 10%) were mixed at 180 °C for 10 min, pelletized, and stored at 80 °C to remove moisture. In a second step, larger quantities of composites were produced using a kinetic mixer, which reduced processing time and helped maintain the fiber quality. Both the mechanical properties of the composites and the fiber structure were examined, showing that adding natural fibers to a polymer can either increase or decrease its tensile strength. Indeed, one challenge in using lignocellulosic fibers to strengthen polyolefins is that the fibers are hydrophilic, while polyolefins are hydrophobic, leading to poor bonding without treatment or coupling agents. Coupling agents like MAPE improve the bond between fibers and the polymer, enhancing the composite’s overall strength. Thanks to the use of MAPE, the tensile strength and Young’s modulus of the composites increased almost linearly with fiber content up to a point. It was additionally demonstrated that these composites could replace polypropylene and its composites, including those reinforced with up to 20% glass fiber.

Cellulose was also successfully isolated by a multi-step process from six agroindustry residues: corncob, corn husk, grape stalk, marc of strawberry-tree fruit, pomegranate peel, and fava pod. Despite the evident morphological differences among the extracted celluloses, results revealed similar compositional and thermal properties [126].

Particleboards, also known as chipboards, are composite materials made from recycled wood fibers and resins, valued for their structural stability and versatile applications in construction and furniture. Flax, one of the oldest fiber crops, has been traditionally used in Egypt for a range of purposes, including linen production, oil extraction, and as animal feed. However, due to a significant decline in flax cultivation—down to about 10% of its former area over the past decade—there is a pressing need to find alternative materials for particleboard production. Citrus branches present a viable alternative. In a study conducted in 2016 at Tanta Flax and Oils Company in Egypt [127], citrus shives were investigated as a substitute for flax shives in particleboard production. The materials used in the study included flax shives, citrus branches, and urea formaldehyde resin. Flax shives were collected after fiber extraction, while citrus branches were obtained from pruning activities. The manufacturing process involved milling the materials, mixing them with urea-formaldehyde resin, layering them, and pressing them under high temperature and pressure. The study findings on the physical and mechanical properties of biocomposites supported the viability of citrus shives as an alternative for chipboards.

Another interesting work carried out by Scaffaro et al. [128] showed that excessive fertilization causes ecological problems due to leaching issues; to solve this problem and promote agricultural sustainability, an innovative green composite for controlled release fertilizers was produced by adding NPK fertilizer flour to a biodegradable polymer with Opuntia Ficus Indica (OFI) particles present abundantly in the Mediterranean Area.

In a study conducted by Gabrielli et al. [129], four different types of lignocellulosic waste—willow, holm oak, palm leaf, and licorice root—were utilized. These raw materials were sourced locally in southern Italy, considering urban waste and commonly available forestry materials, and were incorporated into a polypropylene matrix. The resulting composite displayed significant improvements in tensile strength and modulus, as well as flexural strength and modulus, showing increases of 28% and 110% and 58% and 111% over neat polypropylene, respectively.

Date palm waste (DPW) from Tunisia’s annual date palm pruning was used as a reinforcing filler in a polypropylene (PP) matrix at loadings of 20–60 wt.% [124]. Only grinding was applied to the DPW, ensuring full utilization without residue generation. This study examines the impact of DPW on the composite mechanical properties and water absorption, as well as the effect of adding maleated polypropylene (MAPP) as a coupling agent. Results showed that the reinforcement potential of DPW strongly depends on its aspect ratio and interface quality. The addition of MAPP improved the composite strength and stiffness compared to pure PP, indicating that DPW effectively acts as reinforcement. The difference in reinforcement effects was attributed to improved interface quality between the DPW and polypropylene matrix.

Other researchers from Algeria investigated the mechanical behavior of different composite materials based on palm date waste [130]. Samples are made by thoroughly mixing natural and saturated fibers (leaflet fibers) in a polyvinyl acetate (PVA) matrix with dimensions (160, 15, and 10 mm). Three-point bending tests were performed to determine the modulus of elasticity, and good mechanical properties were obtained in comparison with values obtained from literature. A comprehensive resume of the results described in this paragraph is illustrated in Table 2.

### 4.2. Applications in Packaging

Substances derived from waste and by-products from the agro-food industry are of high interest for the packaging sector. Due to the abundance of different constituents—which were already discussed within this paper and their respective functionalities—there is also a wide range of promising packaging applications in films, coatings, as fillers, or in active and intelligent packaging.

Citrus pectins are film-forming polymers with good barrier capacities against oxygen but only poor water vapor barriers. The mechanical characteristics of pectin films are highly dependent on the pectin used [131]. Yet, the water resistance of pectin films can be increased by coating with polyhydroxyalkanoates (PHA) [132] or the incorporation of several multivalent ions. For instance, the use of Fe^3+^, Cu^2+^, and Ca^2+^ as crosslinking agents could significantly decrease the water vapor permeability and water solubility of pectin-thymol films. Pectin films are used in combination with active substances, as edible or as water-soluble films [133].

Cellulose and its derivatives (e.g., carboxymethyl-, methyl-, ethyl cellulose, cellulose nitrate, -sulphate, -acetate) are widely used in the packaging area. Although the main source of cellulose is wood, it can be extracted from many plant-based sources [134]. Especially cellulose nanomaterials are of major interest, as they usually show improved gas barriers and mechanical stability. The particularity of cellulose nanomaterials, which allows them to exhibit properties such as improved gas barrier performance and enhanced mechanical stability, lies in their highly crystalline structure, nanoscale dimensions, and strong hydrogen bonding interactions. The strong interactions between cellulose nanomolecules provide greater cohesion and stability, making them particularly suitable for applications requiring lightweight yet durable materials. Depending on the nanocellulose material properties, gas permeability may be reduced due to polarity, their dense and ordered arrangement, which minimizes free space, or by their high aspect ratio [135]. They are used in films, coatings, edible films, in active packaging concepts, and as fillers [136]. Nano- or micro-fibrillated cellulose was directly immersed into the polymeric melt through extrusion compounding and then film blowing or cast extrusion [137,138,139] to improve barrier properties, but also as a coating on a biaxially oriented polypropylene/low-density polyethylene (BOPP/LDPE) film to improve its oxygen barrier properties [140].

A high humidity, however, impaired the good oxygen barriers of films, leading to an increasing permeation. Hot pressing of cellulose nanofibril films, as well as a protective coating with latex or polyethylene, was able to reduce this effect [141]. Using nanocellulose as filler in polyvinyl alcohol–banana pseudostem fiber composite films also increased tensile strength (up to 3% filler) and reduced water vapor permeation [142]. Citrus peel waste is another raw material to produce fillers. A filler from citrus limetta peels (5%) was able to increase mechanical parameters (tensile strength, flexural strength, hardness) and the thermal stability of a bioepoxy matrix [143].

Zein, a protein extracted during the processing of corn or rice bran proteins, is an example of proteins suitable to produce films [144]. The production of crystal nanocellulose/corn zein films led to a significant increase in water vapor barriers compared to neat crystal nanocellulose films but only in combination with a crosslinking agent and at a certain crystal nanocellulose/corn zein ratio (15:1 or 10:1), whereas the coating of different crystal nanocellulose/corn zein formulations (3:1–30:1) with a crosslinking agent on BOPP films significantly decreased the oxygen permeability of the film [145].

Waxes, such as the wax of rice bran, a by-product of rice processing, can be used as dispersing aids in compound production [146] but also directly to preserve the freshness of fruit [147]. The wax of rice bran was used as a coating on tomatoes. After a cold storage of 20 days, a reduced weight loss and degradation of chelate-soluble pectin was observed in contrast to uncoated tomato samples [148].

Waste and by-products of the agro-food industry are also promising substrates in the development of active and intelligent packaging concepts. Active packaging, for instance, aims to maintain the quality of perishable food by emitting or absorbing substances like antimicrobials, antioxidants, gases (e.g., O_2_, CO_2_, ethylene), humidity, or light [149,150]. Suitable constituents, powders, or extracts of waste and by-products rich in active substances, like phenols, are incorporated into the packaging. For instance, the integration of active extracts from lemon by-products from a juice-producing plant, citrus peel, grape seed, or date palm fruit waste in active packaging concepts showed antioxidant and/or antimicrobial effects in storage tests with selected foods in comparison to control samples without an active substance [151,152,153,154,155]. Moreover, the integration of orange leaf essential oil in edible gelatin films showed significantly lower microbial growth, oxidative deterioration parameters, and total volatile basic nitrogen (TVB-N) values in cold-stored shrimps, compared to uncoated or gelatin-coated alternatives [156].

Intelligent packaging concepts provide information about the packaged good via data carriers or indicators (e.g., temperature, gases, humidity, pH, pressure) or monitor various parameters (e.g., see indicators, optics, biosensors), which help to draw conclusions about its freshness and quality [157,158]. Natural colorants like anthocyanins or betalains can be used in the design of food freshness indicators. They can be obtained from agro-food wastes and by-products such as purple sweet potato, red cabbage, various berries or red pitaya peel, red beetroot, and red amaranth leaf, and they are able to change colors depending on the pH [159,160]. Anthocyanin-rich extracts of grape skin or date pit were, for instance, used in storage trials using intelligent packaging systems to monitor the freshness of meat or fish [161,162].

Carbon dots (CDs) emerged as novel carbon-based nanomaterials with a size of <10 nm comprising sp2/sp3 hybridized domains and a variety of oxygenated functional groups, such as hydroxyl, carbonyl, and carboxyl groups, as well as amino -NH_2_) groups. Their physicochemical properties resulted in significant chemical versatility and water solubility. Fruit industry wastes, including peels, pomaces, seeds, etc., are carbon-rich substrates that represent sustainable raw materials for the synthesis of functional CDs. Fruit wastes are rich in biomolecules such as polyphenols, polysaccharides, etc., thus, the derived CDs presented interesting functional properties. The synthesis of fruit waste-derived CDs takes place mainly via bottom-up approaches such as hydrothermal, solvothermal, carbonization, microwave, and pyrolysis techniques. Among them, hydrothermal techniques are in the mainstream because of milder reaction conditions, lower reaction times, facile handling, and a higher potential for large-scale production. Peels (e.g., orange, lemon, pomegranate, banana, etc.), including many fruit substrates from the Mediterranean area, shells (e.g., walnut, peanut, etc.), and seeds (e.g., goji berry, apple, neem, etc.) are among the most used fruit-waste substrates used for the synthesis of carbon dots. A summary of techniques for CD synthesis as well as food-waste substrates is presented elsewhere [163,164]. Properties of fruit waste-derived CDs are highly beneficial for food packaging. Previous investigations reporting the incorporation of CDs into a polymer/biopolymer matrix showed increased barrier properties against water, humidity, and gases (e.g., oxygen, carbon dioxide, etc.), as well as UV-blocking properties. Moreover, they present antimicrobial properties and antioxidant activity. Finally, CDs significantly enhanced packing physical characteristics such as mechanical strength, resistance to temperature deterioration, and glass transition temperature of films without altering other properties. As a result, they received more and more applications in packaging for improving food safety and shelf life. However, thorough studies on the toxicity of CDs are necessary to ensure their suitability for food packaging. Research on the use of CDs in food packaging is in full swing, and applications as multifunctional fillers of food packaging materials are continuously explored [163,164].

In order to bring these innovative packaging materials to the market, it is necessary to give due consideration to a number of practical aspects. From an economic standpoint, the production of these bio-based materials must be scalable and cost-effective to compete with traditional plastic packaging. This may entail the procurement of copious agricultural waste from local sources with a view to reducing the costs of raw materials and investments in the development of efficient processing technologies. From a pricing perspective, it is essential to strike a balance between the sustainable premium consumers are willing to pay and the development of competitive packaging solutions. From a legal standpoint, these materials must comply with rigorous food safety standards, ensuring consumer health. Robust testing is necessary to guarantee the stability and functionality of these materials, particularly for components utilized in active and intelligent packaging. Moreover, certifications and compliance with regulatory standards, such as those set forth by the Food and Drug Administration, are essential to gain consumer trust and industry acceptance [165]. In Table 3, an overview of packaging applications derived from agro-food waste and by-products is presented, highlighting their main uses, functionalities, and considerations regarding their performance and potential limitations.

### 4.3. Applications in the Textile Sector

In the EU, textile consumption ranks fourth in terms of negative environmental and climate impacts. According to a 2022 communication from the European Commission [166], global consumption of clothing and footwear is projected to increase by 63% by 2030. Textiles are one of the top five sectors with the highest plastic consumption, accounting for 90% of the global plastic demand by volume [167].

In this context, recycling agricultural waste presents a promising sustainable solution for the textile industry, aligning with principles of circular economy. Agricultural residues can be used to create innovative textiles and contribute to more sustainable coating and dyeing products. This approach addresses one of the major challenges in the textile sector—traditional finishing processes that heavily rely on harmful materials and virgin feedstocks. Moreover, utilizing agricultural residues helps mitigate waste disposal issues and supplies sustainable natural fiber resources to industries like automotive and construction, which benefit from thermal and acoustic insulation materials.

Research on utilizing agricultural waste from the Mediterranean region for renewable textile materials is advancing, with a focus on eco-friendly, cost-effective, and high-performance alternatives to synthetic textiles and conventional dyes. These materials are attracting interest due to their biodegradability and reduced environmental impact compared to synthetic fibers and traditional chemicals, which are prevalent in standard textile production. Notably, residues from olives, grapes, dates, citrus, and nutshells showcase the significant potential of agricultural waste in the textile sector. Waste coming from the olive harvesting and olive oil extraction process is a potential resource for textile finishing.

Various studies [168,169] demonstrate that olive mill wastewater, an effluent generated by the olive oil extraction, could be successfully exploited as a natural dye for wool fabrics, obtaining dark brown shades with high color yield. Literature [170,171] showed that such wastewater could successfully be used for the dyeing of acrylic and polyamide fibers, optimizing an eco-friendly dyeing process. Research has shown that olive pomace, a byproduct of olive oil production, has significant potential as an eco-friendly option for the textile dyeing industry due to its dual purpose. Indeed, the olive pomace contains natural pigments that can color natural fibers, offering a sustainable alternative to conventional synthetic dyes [172], as well as showing effective performance in adsorbing and removing synthetic dyes from textile wastewater, helping reduce environmental pollution while repurposing industrial byproducts [173,174]. Another interesting study [175] also shows the usability of olive tree leaves fallen during olive harvesting in dyeing, assuring fastness and color variety, as well as antibacterial finishing of cotton fabrics for cleaner production. 

Furthermore, studies indicate that common Mediterranean agricultural residues, such as olive pomace or date palm fibers, can be transformed into nonwoven fabrics and composite textiles. Acoustic tests have revealed excellent insulation properties of the olive pomace-derived materials, specifically those based on high surface density non-wovens [176]. Along with favorable chemical and dielectric properties, date palm fibers also show potential for the development of affordable, effective insulation materials [177]. Important studies have also been carried out to assess the use of palm tree date from the Mediterranean region as an effective adsorbent for the removal of dyes from textile wastewater [178,179].

Grape pomace has been found as another effective source of natural dyes for cotton and wool fabrics [180,181], and it has shown excellent UV-protective properties when applied as a dye onto several fabrics (natural and synthetic) with mordants such as potassium alum and ferrous sulfate [182].

Regarding the reuse of orange peel waste for sustainable textile production, recent studies have explored how cellulose fibers derived from citrus waste offer eco-friendly, vitamin-enriched textiles [183]. These materials are used in apparel and boast benefits like skin nourishment, anti-microbial and mosquito-repellent properties, and a reduced carbon footprint, making them a promising alternative to traditional fabrics. Fibers from orange residues are very versatile, including applications in dyeing and even in textile wastewater treatment [184].

The use of nutshells as a dye source was evaluated by different studies. Among them, it is interesting to mention the use of the shells of peanuts, cashew nuts, coconuts, and macadamia nuts as a possible dye source for the coloration and multifunctional finishing of wool fabric [185]. Exploiting the extracts containing quinone and phenolic compounds, the research shows effective dyeing and fastness properties as well as UV protection (even in the absence of mordants). Another recent study has also developed a method to extract lignin with antioxidant properties from almond by-products, offering potential applications for the textile industry [186]. Lignin could be incorporated into textile fibers to create fabrics with enhanced durability and protective qualities, such as both resistance to UV degradation and microbial growth.

The following table (Table 4) summarizes the agricultural wastes and their potential uses, along with key findings and the corresponding references.

## 5. Recovery by Bioconversion

### 5.1. Production of Biopolymers by Microorganisms

Fruit-related agricultural waste can be used to produce polymers through biotechnology. Many fruit residues, like peels, seeds, and pulp from citrus, grapes, and other fruits, are rich in sugars, starches, and cellulose, which serve as valuable raw materials for biopolymer production. Using biotechnological processes, these natural compounds can be transformed into biopolymers such as polylactic acid (PLA), polyhydroxyalkanoates (PHA), and other biodegradable plastics [187]. This approach is not only sustainable but also offers an efficient method to recycle agricultural waste, contributing to circular economy goals. The resulting biopolymers are commonly used in packaging, textiles, and even biomedical applications due to their biodegradability and lower environmental impact.

Microorganisms act as small factories for producing various polymers throughout their growth cycles. These microbial polymers are highly valued due to their biodegradability, biocompatibility, nontoxicity, and adaptability to diverse substrates.

Monomers that can be obtained by biotechnology are lactic acid, succinic acid, glutaric acid, adipic acid, 1,2,4-butanetriol, 2,5-Bis(hydroxymethyl)furan-3-carboxylic acid (MCDCA), itaconic acid, 2,5-Furandicarboxylic Acid (FDCA), malate, cadaverine, terephthalic, glucaric acid, cis,cis-muconic acid, 1,3-PDO, and malonic acid [188]. The corresponding biopolymers can be used in food, medicine, wastewater treatment, biofuel production, packaging, agriculture, and cosmetics [189].

Polymers that can be obtained through this route can be highly attractive from an industrial and applicative point of view. Indeed, biopolymers such as alginate, cellulose, cyanophycin, levan, polyhydroxyalkanoates, xanthan, poly(lactic acid), and poly(γ-glutamic acid) can be obtained from different microorganisms like *Aureobasidium pullulans*, *Acetobacter xylinum*, *Bacillus thermoamylovorans*, and *Cupria-vidusnecator*. The class of polyhydroxyalcanoates (PHA) is very extended, including homopolymers and various copolymers that, over time, can be produced with better reliability [189]. Several commercial PHA materials are available on the market.

Pullulan (PUL) is a biopolymer produced by strains of the polymorphic fungus *Aureobasidium pullulans* as an extracellular, water-soluble polysaccharide [190] and has a wide range of applications in the cosmetic and biomedical fields. Interestingly, Teno et al. [191] developed a pullulan-based electrospun beauty mask that was modified by dry powder impregnation with chitin nanofibrils-nanolignin complexes containing glycyrrhetinic acid. The polymers obtained by microorganisms are extensively used in various applicative sectors like food, packaging, medicine, pharmaceutics, cosmetics, wastewater treatment, and biofuel production [192]. Despite its evident advantages, the biopolymer market is still facing several hurdles [193]. It was recently evidenced that the main drawback limiting the development of these polymers is the high production cost and low efficiency of the microbial strains.

### 5.2. Bioconversion Exploiting Insects

Insects are the greatest example of biodiversity on Earth, playing a highly diverse role within ecosystems [194]. Despite often being seen as pests, insects provide many crucial ecological services, including the bioconversion of organic by-products [195]. Bioconverter insect species are particularly relevant as, during their larval stages, they can consume a wide range of vegetable and animal decaying organic matter, rapidly grow, and gain nutrients that accumulate in their biomass, rich in proteins, lipids, and chitin, that can be extracted and utilized as food ingredients or for other applications, creating high-value, marketable products [196]. As a result of the bioconversion process, several food waste and by-products can potentially be valorized and repurposed as new high-biological value products instead of being conventionally discarded, gaining new value [196]. In recent years, *Hermetia illucens* (Diptera: Stratiomyidae), commonly known as the Black Soldier Fly (BSF), has attracted special attention among bioconverter insects, as it can voraciously feed on a wide range of organic substances [197], including agri-food chain waste [198], distilled grain by-products like spent grain [199], manure [200], catering, cafeteria, and supermarket waste [201,202], and many others. In optimal environmental (27 °C of temperature and 70% of relative humidity) and substrate (70% of humidity) conditions, *H. illucens* can consume the substrate in around 10–15 days, reducing it until the 70–80% (wet way), bio-accumulating proteins and lipids, whose concentration and quality in terms of amino acids and fatty acids strictly depend on the feeding substrate [203,204,205]. This biomass, if derived from larvae fed on specific vegetal organic by-products, can be used for animal feed (pets, insectivorous animals, poultry, pigs, fish) (EU Regulations 2017/1017 and 2021/1372); on the contrary, larvae fed on other waste can be used exclusively for research purposes or non-feed applications, for example, lipids can be used for high-quality biodiesel production [200] or protein can be used for mulching purposes [206].

**Figure 7:**
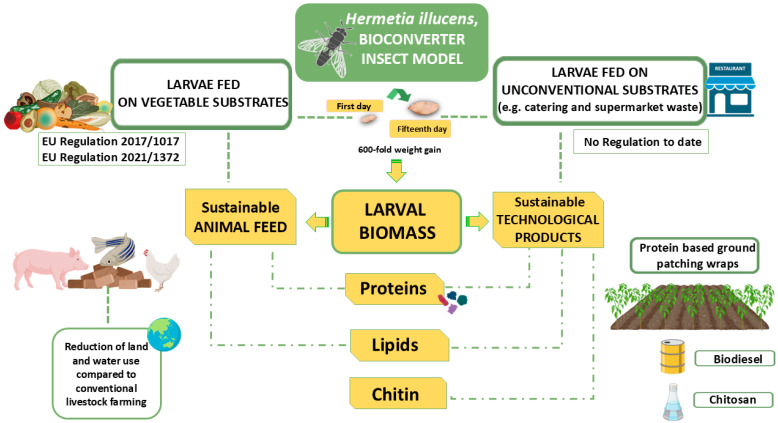
Valorization of organic waste by insect bioconversion. Insect larvae, particularly the Diptera *Hermetia illucens*, can be reared on organic substrates that are bioconverted in larval biomass, rich in protein, lipid, and chitin.

Depending on the starting substrate, which in any case needs to be authorized by current European regulations, the products resulting from the bioconversion process can be used in animal feed or for innovative and sustainable technological solutions as shown in Figure 7.

In this context, special attention should be given to agro-industrial waste, a substrate allowed as feed for insects, which can subsequently be utilized in feed applications. Given that one-third of global agricultural and food production is wasted and considering that insects can be reared on former foodstuffs, they represent a critical solution for reducing food waste by converting it into valuable products, not only for animal feed and human food and bioenergetic fields, as previously mentioned, but also for application in biomedical, pharmaceutical, and agricultural fields. Indeed, another key product of the insect rearing on waste and by-products is chitosan. Chitosan can be produced, by a deacetylation process, from chitin of pupal exuviae and dead adults of insects, the only side streams of the bioconverter insect rearings, that are an example of a perfect circular economy process, with zero waste [207,208]. Indeed, also insect frass (composed of larval excrements, larval exuviae, and unconsumed feedstock) finds application in agriculture [209]. Chitosan, a biodegradable, eco-compatible, atoxic, anti-microbial polymer, can be employed in several industrial applications: cosmetics [210], pharmaceuticals (drug delivery, wound healing) [211,212], environmental (anti-microbial against plant pathogens, plant immune defense promoter, wastewater treatment) [213,214,215], and food (packaging, food shelf-life booster) sectors [216,217,218,219,220].

Additionally, the high potential in reducing waste by bioconverting it into high-value products, another key advantage of using insects in feed and food, replacing traditional protein and lipid sources, is the reduction in land and water use in these breedings, as well as the greenhouse gas emissions, compared to traditional livestock farming [200].

Furthermore, insect protein can be an ideal substitution or integration also of nutritional supply deriving from conventional crops, like soy, which are widely used as animal feed but compete with the use of land for human food production, or deriving from fish meal, depleting the oceans and disturbing the delicate marine ecosystem.

The world’s growing population, along with the increasing demand for food and agricultural land, the overproduction of food waste, the depletion of natural resources, and the pressing climate change, has led to a global awareness of the need for new, alternative sources of food for both humans and animals. Bioconverter insects can offer their potential in a circular economy from an economic perspective, as they can convert organic waste into insect biomass that can be used as feed for livestock or even as food for humans. The vast diversity of insect species and the wide variability in their composition further highlight their potential.

In this context, insects offer a viable and sustainable source and a forward-thinking, marketable solution for waste management.

### 5.3. Mediterranean Food Wastes Valorized Through Their Transformation for Energetic Purposes

**Figure 8:**
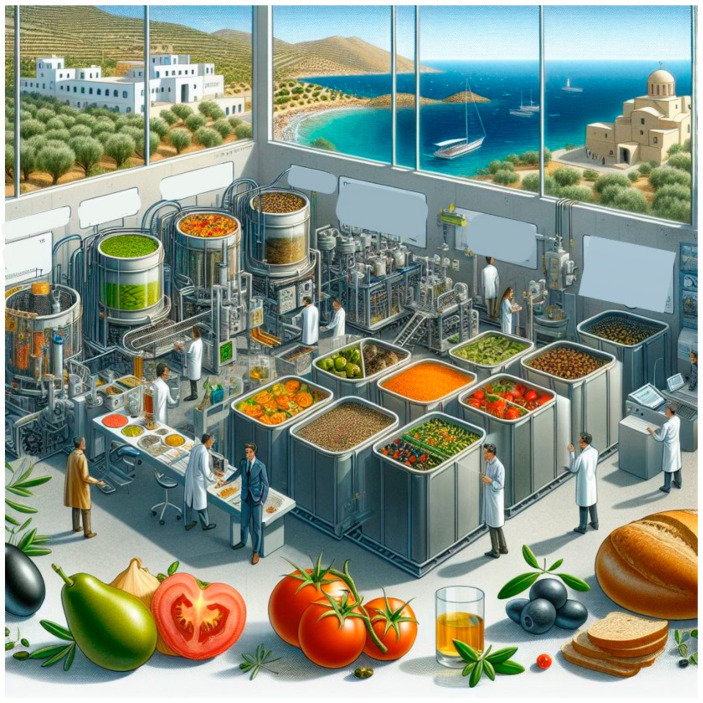
Ideal representation of the food waste valorization for energetic purposes.

The valorization of Mediterranean waste materials for energy purposes (ideally illustrated in Figure 8) presents significant opportunities for sustainable waste management and bioenergy production [221]. Several studies have explored different approaches to converting agricultural and food waste into valuable energy resources, including biofuels, biogas, and electricity [222,223].

Manara et al. [224] focused on the valorization of by-products from Mediterranean agri-food processes, such as olive oil, wine, and fruit, which pose disposal challenges due to their high organic content and potential health and safety concerns. These wastes, particularly under the Mediterranean climate, foster microbial growth. By utilizing pyrolysis after pre-treatment and recovery of valuable precursor materials like lignin and pulp, these wastes can be transformed into biofuels, chemicals, and carbon-based materials. Pyrolysis experiments, including thermal degradation kinetic studies, demonstrated the feasibility of these processes with good agreement between experimental data and theoretical models. This research highlights the potential for transforming Mediterranean agri-food wastes into valuable energy and material resources through efficient thermo-chemical processes. DeMichelis et al. [225] emphasized the importance of effectively managing agro-food waste (AFW), which presents significant environmental, social, and economic challenges, particularly in Italy, one of the leading agricultural producers and food processors in the EU. The authors highlighted the need to treat AFW not just as a disposal issue but as a resource for high-value compounds and bioenergy. Their review provides an overview of AFW management processes according to circular economy principles, exploring available technologies at different Technology Readiness Levels (TRLs), with a particular focus on the Italian context. The review describes industrial-scale and pilot-scale plants, as well as emerging biological approaches aimed at converting AFW into valuable compounds and energy. This approach promotes the inclusion of AFW within a circular economy framework, enhancing the sustainability of waste management by considering these materials as valuable resources. Antonopoulou et al. [226] investigated the valorization of food waste at the municipality level, focusing on electricity production and methane generation. After the heat drying and shredding, food waste was extracted with warm water, producing a liquid extract and a solid residue. The extract, rich in soluble chemical oxygen demand, was used in a microbial fuel cell (MFC) for electricity production, while the solid residue underwent anaerobic digestion to produce methane.

The study found that energy recovery from food waste is promising, with optimized operational conditions leading to efficient bioenergy outputs, making it an appealing strategy for urban waste management and renewable energy generation.

In a similar vein, an integrated engineering system [227] was developed to produce bioenergy from food waste, with a focus on cost-effective and active enzymatic pre-treatment. This process involved the use of a fungal mash, produced in situ from food waste, to hydrolyze the waste and generate glucose. The glucose was then fermented to produce bioethanol, and the remaining residue was anaerobically digested for biomethane production. The system achieved a significant reduction in food waste (90% total solid reduction) and generated bioethanol and biomethane, offering a dual bioenergy production pathway. The economic feasibility and technical viability of this integrated system were demonstrated through cost-benefit analysis, underscoring the potential for large-scale implementation of such waste-to-energy processes.

## 6. Efficiency Gains Through Digitalization

Recent developments in digitalization influence all areas of daily life. Especially technologies such as sensors combined with machine learning can ubiquitously collect and analyze data for real-time prediction and optimization of processes. This can also improve residue and side-stream processing.

Next, Section 6.1 defines important technologies in this context, namely artificial intelligence, machine learning, deep learning, and the internet-of-things, and describes their application for side-stream processing. Afterwards, Section 6.2 elaborates on the potential of digital twin technology for analysis in side-stream processing. Lastly, Section 6.3 summarizes how digital technologies can support the integration of systems and, through this, a holistic analysis within production processes.

### 6.1. Artificial Intelligence (AI), Machine Learning, and Internet-of-Things

Recent advancements in information technology, particularly in artificial intelligence, machine learning, and deep learning, are transforming various sectors, including agriculture. These technologies are not only enhancing productivity but also playing a crucial role in promoting sustainable practices, such as the reuse of agricultural by-products and minimizing waste in food production [228].

Artificial intelligence (AI) refers to systems that perform tasks typically requiring human intelligence, such as decision-making, pattern recognition, and process optimization [229]. Within AI, machine learning is a specialized approach that uses algorithms to identify patterns in data and make predictions, which is particularly useful for analyzing large datasets. Machine learning (ML) techniques can be broadly categorized into three main types: (1) Supervised Learning, (2) Unsupervised Learning, and (3) Reinforcement Learning, each with its own advantages and disadvantages depending on the availability of annotated data [230]. Supervised Learning employs labeled data, where each input is associated with a known output, to predict outcomes for new, unseen data. This approach is commonly applied in agriculture for tasks such as crop yield prediction and disease detection, facilitating effective forecasting and classification. In contrast, Unsupervised Learning analyzes unlabeled data to uncover patterns or structures, focusing on clustering data points based on similarities. This method is particularly useful for anomaly detection, aiding in the identification of unexpected behaviors in agricultural data. Lastly, Reinforcement Learning involves an agent interacting with its environment to maximize rewards through trial and error, making it valuable for optimizing decision-making processes like resource allocation in farming operations. Collectively, these ML techniques support a wide range of applications in the agricultural sector, enhancing practices and decision-making through improved prediction, classification, clustering, and anomaly detection. Li et al. demonstrate the effective use of ML to enhance circularity by optimizing biogas production from food waste [231]. The study identifies key input variables that significantly impact biogas yields, including feedstock characteristics, environmental conditions, and operational parameters. By applying ML models, the researchers accurately predicted biogas output and identified optimal conditions to maximize production efficiency.

Deep learning, a subset of ML, involves artificial neural networks that can model complex, nonlinear relationships in data, making it especially effective in tasks such as image and audio recognition [232]. Deep learning (DL) is considered an advancement over traditional machine learning algorithms, significantly enhancing both efficiency and speed in addressing complex problems. This enhancement is largely due to the increased “depth” (complexity) of its models. One of the key advantages of deep learning is its ability to automatically extract features, enabling it to effectively identify high-level patterns within large datasets [233].

In modern agricultural production, sensors play a critical role in optimizing processes. Through the integration of the Internet-of-Things (IoT), sensors can be connected and integrated with computational resources [234], hence providing real-time monitoring. Using AI, the analyzed information can build the foundation for intelligent control over various aspects of production, contributing to sustainability by enabling better resource management and reducing waste through improved monitoring and decision-making.

IoT combines advancements in miniaturization and connectivity, making it possible for devices equipped with sensors to collect and act on environmental data [235]. In agricultural production, these sensors monitor key parameters such as temperature, gas concentrations, or humidity, among others. By continuously collecting data, sensors ensure that the by-products generated during food processing—such as organic waste—are handled in an optimal manner, supporting the efficient conversion into renewable resources like biopolymers. Further, the quality of the by-products can be predicted and, hence, the set of possibilities for reuse.

Sensors integrated within production lines can monitor the quality and status of materials, allowing for precise adjustments that enhance the reuse of such by-products. For example, gas sensors measure concentrations of gases such as CO_2_ or hydrogen sulfide (H_2_S), which are key indicators in processes [236]. These measures enable producers to maintain optimal conditions.

Additionally, biosensors—which use biological elements such as enzymes or nucleic acids—offer a valuable tool in monitoring organic by-products. These sensors can detect specific chemical markers, helping to evaluate the current condition [237]. By providing real-time feedback on the quality of these materials, biosensors contribute to the efficient reuse of waste in a safe and controlled manner.

Through the integration of sensor technology, agricultural producers can optimize the management of by-products, contributing to a more sustainable production cycle. By monitoring environmental conditions and ensuring the proper reuse of materials, sensors help drive innovation and efficiency in agricultural production, enabling a circular approach to resource use.

Additionally, Generative AI, as mainly known from ChatGPT, can help to generate new content, such as text, pictures, videos, etc. Generative AI can help to accelerate the circular economy by optimizing supply chains and identifying opportunities for resource reuse. It can also assist in designing innovative products that minimize waste and maximize the use of by-products or side streams. Furthermore, AI can support decision-making by analyzing vast amounts of data to propose efficient ways to recycle materials and reduce the environmental impact. However, to the best of the authors’ knowledge, Generative AI has not been applied in such use cases so far [238].

### 6.2. Digital Bio-Physical Twins

With the development and digitalization towards Industry 4.0, the concept of digital twins has found its way into the industry. A digital twin is the digital representation of an active, unique product (a real device, object, machine, service, or intangible asset) or a unique product-service system consisting of a product and an associated service [239].

The digital twin enables the integration of real-time data and simulations, allowing continuous monitoring, analysis, and optimization throughout the life cycle of the product or service.

A core concept is that, especially for food processing but also other bio-based products, biological and physical processes often proceed independently of machinery actions, as in the case of yogurt fermentation, where bacterial activity plays a crucial role. Traditional digital twins focus heavily on machine data [240], but digital bio-physical twins also factor in microbiological changes, chemical reactions, and variations in raw materials, like seasonal differences in milk [241].

However, a digital bio-physical twin has additional specific requirements compared to digital twins to produce material goods, as these cannot be based solely on the processing steps due to the variability of the raw materials but must also consider the chemical, physical, or (micro)biological product/process properties [241]. These digital twins provide relevant information (i) for real-time process control and troubleshooting and (ii) for optimizing processes regarding consistency, performance, and sustainability.

Digital bio-physical twins can be used to optimize production planning or form the basis for autonomous systems, but they are also useful in optimizing sustainability and side-stream or residue processing. Digital bio-physical twins can also simulate the effects of packaging on the shelf life of food. A digital food twin could even serve as a replacement for storage tests that are commonly used to predict shelf life [241].

### 6.3. Circularity

Traditionally, the valorization of agricultural waste has relied on physical and chemical methods for extraction and processing, often requiring extensive trial-and-error to achieve optimal performance. While effective, these methods frequently lead to significant energy consumption, the use of hazardous chemicals, and a lack of specificity in targeting valuable compounds. Consequently, they can yield lower returns and increase environmental impact. The integration of digital technologies, including AI, IoT, and Digital Bio-Physical Twins, can significantly enhance these biological methods. For instance, various parameters and conditions, along with suitable biological pre-treatment and treatment processes, can be optimized and automated to ensure improved performance [242]. AI models, such as machine learning algorithms and deep neural networks, can be trained to predict the most efficient extraction methods based on input parameters such as temperature, pressure, and time. These predictions rely on historical data, real-time sensor inputs, and the properties of agricultural by-products like fruit peels or plant residues. For example, olive pit residues contain valuable phenolic compounds, and AI can determine the best extraction conditions to maximize yields while preserving the quality of bioactive compounds. Additionally, fibers from palm leaves could be transformed into construction panels or biodegradable packaging through AI-optimized extraction.

When implementing AI-based approaches, several key steps are necessary, including data collection, preprocessing, model training, evaluation, and deployment. Each stage is essential to ensure the overall effectiveness and reliability of the solution [243]. The first step is to gather data from agricultural operations using IoT devices. By deploying sensors to monitor food waste production, farmers can track the quantity and types of waste generated in real-time. This data serves as the foundation for further analysis. AI algorithms can analyze this data to identify patterns, predict potential quality changes, and optimize resource allocation. This holistic analysis can also pinpoint bottlenecks in the production process, ultimately improving both productivity and sustainability.

To facilitate real-time monitoring and intervention, dashboards can be utilized to display key performance indicators and alerts in case of anomalies, providing a comprehensive view of the production process. These dashboards offer a detailed snapshot of several parameters and alerts for any deviations from optimal conditions, enabling producers to respond promptly to potential issues.

The concept of digital bio-physical twins complements this approach by providing detailed digital representations of not only the physical machinery but also the biological and chemical processes involved. These digital twins integrate real-time data and simulations to provide a holistic view of production processes, which is crucial in agricultural contexts where biological factors significantly impact the final product quality and sustainability. Utilizing digital twins can help analyze the lifecycle of products and identify the properties of by-products and their potential.

By combining IoT-enabled monitoring and AI-based analysis with digital bio-physical twins, producers can simulate and optimize the entire lifecycle of their products, including raw material variations and the processing of by-products. This dual focus on physical and biological aspects enables precise adjustments in real-time, improving the efficiency of by-product reuse and ensuring minimal waste. For instance, the integration of digital twins allows producers to optimize fermentation processes, maximizing the yield of valuable secondary products while maintaining consistent quality.

**Figure 9:**
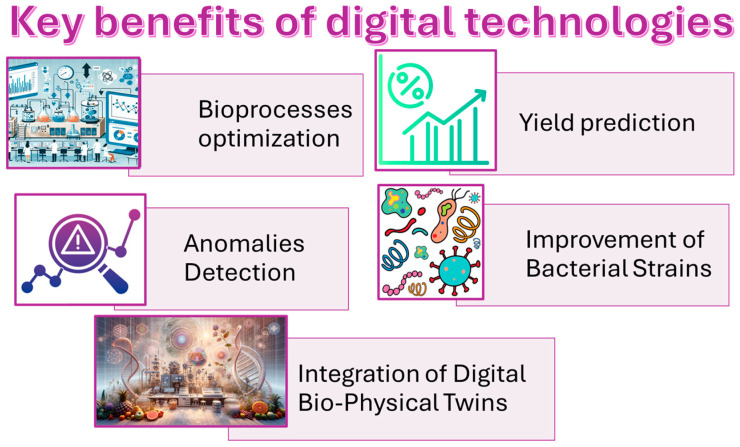
Key benefits provided by digital technologies.

However, the complexity and variability of raw materials, their properties, and the limited shelf life of raw food materials and their products pose challenges to application potential. Additionally, the constantly changing systems, processes, and knowledge about biological or chemical processes for food production and processing necessitate continuous improvement of corresponding digital twins. A lack of reliable physicochemical data from these processes is another major obstacle to using modeling and simulation tools. Furthermore, the kinetics of biological and chemical processes must be understood and made calculable through physical models, which can be integrated into the context of physics-informed machine learning, serving as the basis for the learning process of artificial neural networks. Process models can be utilized to estimate energy and material requirements, as well as the expected process yield during food processing, for example, determining energy requirements by simulating process steps based on previously collected data. The use of bioconversion for circularity can be further enhanced through the integration of digital technologies. Specifically, producers can recover valuable compounds from waste streams, reducing waste and generating new revenue streams. This approach contributes to a more circular and sustainable agricultural production model, minimizing the industry’s environmental footprint while enhancing its economic viability. Therefore, digital technologies provide several key benefits (Figure 9):

- Optimization of Bioprocesses: AI, particularly machine learning algorithms, can analyze experimental data and adjust process conditions in real-time, maximizing enzymatic decomposition efficiency and improving yields of valuable compounds.
- Yield Prediction: AI can help predict the quantities of extracted compounds based on the types of agricultural residues, cultivation conditions, and processing parameters, enabling producers to anticipate available raw materials and effectively plan the production of renewable products.
- Early Detection of Anomalies: The use of AI and IoT sensors to monitor processes in real-time allows for the detection of anomalies or inefficiencies in bioprocesses, preventing production losses and enhancing process stability.
- Improvement of Bacterial Strains: Through genetic data analysis and scenario simulations, AI can identify genes of interest to enhance bacterial strains, increasing their effectiveness in releasing bioactive compounds.
- Integration of Digital Bio-Physical Twins: Digital Bio-Physical Twins facilitate the creation of virtual models of biological processes. These models can simulate and predict outcomes based on various inputs, enabling better decision-making and process optimization.

## 7. Economic Feasibility, Scalability, Regulatory, and Policy Barriers

Despite the significant potential of valorizing Mediterranean fruit by-products into renewable materials, several challenges hinder their practical implementation. One of the primary barriers is the variability in the composition and quality of agro-food waste, which can affect the reproducibility and consistency of the final products. Seasonal availability and geographic dispersion of these by-products further complicate the logistics of collection and processing [244]. Additionally, the lack of standardized protocols for the extraction and functionalization of bio-based materials poses technical difficulties, limiting their widespread adoption [245].

The economic feasibility of valorization processes is a key factor for their successful implementation. The scalability of these technologies is another critical issue, as many laboratory-scale processes face technical and economic challenges when transferred to industrial production. Moreover, the cost-effectiveness of bio-based materials needs to be carefully evaluated in comparison to conventional materials. Conducting comprehensive life cycle assessments (LCA) and techno-economic analyses can provide valuable insights into the long-term viability of these solutions [246].

Regulatory frameworks play a crucial role in the adoption of renewable materials derived from agricultural waste. Compliance with food contact safety regulations, such as those set by the European Food Safety Authority (EFSA) and the Food and Drug Administration (FDA), is essential for market approval as described in the previous sections. Differences in regulatory standards between countries may pose further obstacles for companies aiming to enter international markets. Policies promoting circular economic practices, along with financial incentives and certification schemes, could facilitate the adoption of these materials. As underlined, to demonstrate the practical potential of valorization strategies, real case studies and quantitative data are essential. Recent initiatives have shown promising results. For instance, the Green Farm Project [247] aims to transform agricultural waste into useful resources; olive tree prunings are used to produce pellets for domestic heating, reducing the need for fossil fuels. Moreover, a project promoted by ENEA (Italian National Agency for New Technologies, Energy, and Sustainable Economic Development) called PROVIDE [248] uses sustainable extraction techniques to obtain bioactive compounds from agro-food waste. These compounds can be used in the food, nutraceutical, and cosmetic sectors. The use of citrus peel waste in biodegradable packaging films or grape seed extracts in active packaging applications achieving good results in oxygen permeability compared to conventional plastic films has been performed in southern Italy [249].

## 8. Conclusions

Through a bibliographic survey, key sources of Mediterranean fruit by-products and waste were analyzed. Despite their abundance and high potential for valorization, most of these by-products have been studied and developed primarily at the laboratory scale, with limited systematic applications at the industrial level. Various valorization methods have been identified, including: (1) extraction to obtain valuable antimicrobial and antioxidant compounds for cosmetics, nutraceuticals, and pharmaceuticals, leveraging their bioactive properties; (2) transformation into composite materials and fibers suitable for packaging, textiles, and personal care products; and (3) biotechnological conversion into biopolymers and innovative materials, such as through insect rearing and related bioconversion processes. The methods of utilizing waste presented here are just a few of the many possible approaches. However, they deserve attention from the scientific community as they potentially offer innovative solutions compared to current practices. This represents the main contribution that this review would like to offer to this research area.

**Figure 10:**
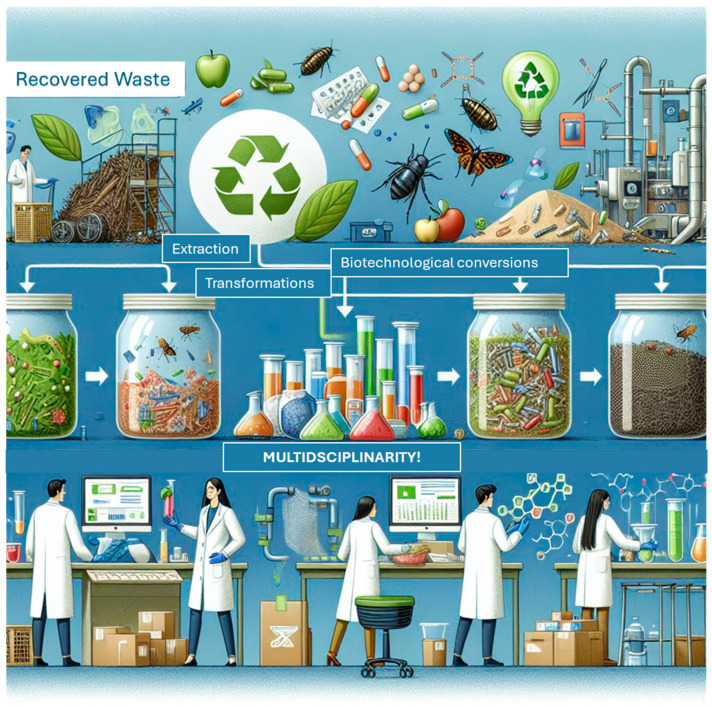
Multidisciplinary approach of the valorization of Mediterranean fruit by-products and waste.

Moreover, to enhance efficiency and cost-effectiveness, a multidisciplinary research approach integrating these valorization methods is crucial, alongside the implementation of digital tools to facilitate their adoption (Figure 10).

Comparing these findings with previous reviews on agro-industrial waste valorization in the Mediterranean area, similar challenges and opportunities emerge. Studies such as those by Galanakis [250] and Mirabella et al. [251] highlight the significant untapped potential of agro-industrial by-products but emphasize the need for scalable industrial processes and policy support. Likewise, Gullón et al. [252] discuss the role of biorefinery approaches in the Mediterranean context, underscoring the importance of integrating waste valorization into a circular bioeconomy framework. Our review builds upon these insights by not only reaffirming the potential of extraction, composite material production, and biotechnological conversion but also proposing a novel approach to circularity: the development of bio-based, biodegradable smart packaging for fruits, incorporating agricultural waste from the same plant.

This strategy, which will be explored in future research as part of the PLAMINPACK project, aligns with ongoing efforts to promote circularity and engage end users in sustainable innovation. By leveraging the intrinsic antimicrobial and antioxidant properties of agricultural residues, along with biopolymer-based protective coatings, this approach could significantly extend fruit freshness and enhance sustainability in the Mediterranean agri-food sector. Further collaboration between researchers, policymakers, and industries will be essential in advancing these strategies and fostering a resilient bio-circular economy for the region.

## Figures and Tables

**Table 1:** Typical composition of olive pomace, a byproduct of olive oil extraction. The composition varies depending on factors such as olive variety, processing methods, and extraction conditions.

Component	Weight Percentage (%)
Moisture	40–70
Residual oil	2–8
Total Organic Matter	85–95
Crude Fiber	25–45
Proteins	3–10
Ash	2–5
Polysaccharides and Sugars	20–40
Lignin	10–30
Polyphenols	1–5
Minerals	Traces

**Table 2:** Resume of the Section 4.1 main information.

Material Used as Filler	Matrix	Shape of the Reinforcement	Key Findings
Hazelnut Shell (HS, HSP)	PLA (Polylactic Acid)	Powder	Increased stiffness but reduced tensile strength; improved UV resistance; semi-industrial production feasible; potential for 3D printing.
Walnut Shell (WSP)	Fossil-based thermoplastics	Powder	Often used in durable applications like furniture and automotive.
Almond Shell (ASP)	Biobased thermoplastics	Powder	Increased mechanical properties
Citrus Pomance	Polysaccharide-based biocomposites	Fibers of pectin and cellulose	Used for biodegradable mulching films; enhanced biodegradation
Orange Tree Pruinings	BIO-PE (Biopolyethylene)	Fibers	Improved tensile strength and Young’s modulus; potential PP replacement
Citrus Branches	Urea-formaldehyde resin	Shives	Viable substitute for flax in particleboard production
Opuntia Ficus Indica (OFI)	Biodegradable polymers	Particles	Green composite for controlled-release fertilizers
Lignocellulosic Waste	Polypropylene (PP)	Willow, holm oak, palm leaf, licorice root	Increased tensile and flexural strength
Date Palm Waste (DPW)	Polyvinyl acetate (PVA)	Leaflet fibers	Good mechanical properties confirmed via three-point bending tests

**Table 3:** Agricultural wastes and their potential uses in packaging applications.

Derived Substance	Application	Function	Key Findings
Pectin (citrus waste)	Films, coatings, edible films	Oxygen barrier, reduced water solubility with crosslinking	Poor water vapor barrier, improved with PHA or multivalent ions
Cellulose and derivatives (plant waste)	Films, coatings, fillers, active packaging	Improved gas barriers, mechanical stability	Nanocellulose improves barrier properties but is sensitive to humidity
Citrus peel waste	Filler for bioepoxy	Increased mechanical strength and thermal stability	Used in biocomposites
Zein (corn or rice bran proteins)	Films, coatings	Water vapor and oxygen barrier (with crosslinking agents)	Improvements only with crosslinking and specific composition ratios
Waxes (rice bran wax)	Coatings	Fruit freshness preservation, reduced weight loss	Used for tomato storage
Extracts from waste (lemon, grape seed, date)	Active packaging	Antimicrobial and antioxidant properties	Extends food shelf life
Natural colorants (purple sweet potato, red cabbage, berries, beetroot)	Intelligent packaging	Freshness indicators with pH-based color change	Used to monitor meat and fish freshness

**Table 4:** Agricultural wastes and their potential uses in textile applications.

Agricultural Waste	Application	Key Findings
Olive Mill Wastewater	Natural dye for wool fabrics	Olive mill wastewater can successfully dye wool fabrics dark brown with high color yield, also effective for acrylic and polyamide fibers in eco-friendly dyeing.
Olive Pomace	Dye for textiles & adsorbent for wastewater	Olive pomace contains natural pigments for dyeing natural fibers and can adsorb synthetic dyes, reducing environmental pollution while repurposing industrial byproducts.
Olive Pomace & Date Palm Fibers	Nonwoven fabrics and composite textiles	Olive pomace and date palm fibers used to create nonwoven fabrics with excellent insulation properties, also showing potential for composite textiles.
Date Palm Fibers	Adsorbent for textile wastewater	Date palm fibers effectively adsorb dyes from textile wastewater, helping in pollution control.
Grape Pomace	Natural dye for cotton and wool	Grape pomace used as a natural dye for cotton and wool, providing UV protection and effective dyeing properties with various mordants.
Nutshells (Peanut, Cashew, etc.)	Dye source for wool fabrics	Nutshells (peanut, cashew, coconut, macadamia) used as a dye source for wool fabrics, providing effective coloration, UV protection, and fastness without mordants.
Almond By-products	Lignin extraction for textile industry	Lignin extracted from almond by-products can be incorporated into textile fibers, enhancing durability, UV resistance, and microbial growth prevention.

## Data Availability

Not applicable.
